# MFA-Net: Motion Feature Augmented Network for Dynamic Hand Gesture Recognition from Skeletal Data †

**DOI:** 10.3390/s19020239

**Published:** 2019-01-10

**Authors:** Xinghao Chen, Guijin Wang, Hengkai Guo, Cairong Zhang, Hang Wang, Li Zhang

**Affiliations:** 1 Department of Electronic Engineering, Tsinghua University, Beijing 100084, China; chen-xh13@mails.tsinghua.edu.cn (X.C.); zcr17@mails.tsinghua.edu.cn (C.Z.); chinazhangli@tsinghua.edu.cn (L.Z.); 2 AI Lab, Bytedance Inc., Beijing 100086, China; guohengkai@bytedance.com; 3 Beijing Huajie IMI Technology Co., Ltd, Beijing 100193, China; wanghang@hjimi.com

**Keywords:** skeleton, gesture recognition, recurrent neural networks, feature augmentation

## Abstract

Dynamic hand gesture recognition has attracted increasing attention because of its importance for human–computer interaction. In this paper, we propose a novel motion feature augmented network (MFA-Net) for dynamic hand gesture recognition from skeletal data. MFA-Net exploits motion features of finger and global movements to augment features of deep network for gesture recognition. To describe finger articulated movements, finger motion features are extracted from the hand skeleton sequence via a variational autoencoder. Global motion features are utilized to represent the global movements of hand skeleton. These motion features along with the skeleton sequence are then fed into three branches of a recurrent neural network (RNN), which augment the motion features for RNN and improve the classification performance. The proposed MFA-Net is evaluated on two challenging skeleton-based dynamic hand gesture datasets, including DHG-14/28 dataset and SHREC’17 dataset. Experimental results demonstrate that our proposed method achieves comparable performance on DHG-14/28 dataset and better performance on SHREC’17 dataset when compared with start-of-the-art methods.

## 1. Introduction

Hand gesture provides an efficient and natural way for human–computer interaction (HCI) due to its flexibility and expressiveness. Hand gesture recognition has great potentials for applications in sign language recognition, remote control and virtual reality and has attracted great research interest in past decades [1,2,3,4,5,6,7,8,9,10,11,12,13,14]. Generally, hand gesture recognitions are categorized into static hand gesture recognition [3,4,15] and dynamic hand gesture recognition [5,6,7]. Static gesture recognition predicts the configuration or posture of the hand from a single image, while dynamic hand gesture recognition aims to understand what a hand sequence conveys. In this paper, we focus on dynamic hand gesture recognition. It remains a challenging task due to high intra-class variance because the way of performing a gesture differs from person to person.

Existing methods on dynamic hand gesture recognition usually take RGB images and depth images [16,17] as input. Some of them use multi-modal input including IR images [6] or audio stream [7]. Recent progresses on hand pose estimation [18,19,20,21,22,23,24,25] enable the acquisition of more accurate hand skeletons in real-time. Commercial sensors such as LeapMotion [26] and Intel Realsense Camera [27] can capture the hand poses with reasonably good quality. Therefore, the research on dynamic hand gesture recognition from 3D hand skeleton sequences has been greatly promoted.

De Smedt et al. [28] proposed a skeleton-based approach for dynamic hand gesture recognition and demonstrated its superiority over depth-based approaches. Temporal pyramid representation is utilized to model temporal information. Similarly, most recent methods for skeleton-based dynamic hand gesture recognition are based on hand-crafted features [29,30,31]. The temporal information is not fully exploited. Another family of solutions utilizes recurrent neural networks (RNN) to process the input hand skeleton sequences and predict the gesture class [32,33]. However, these methods only treat the raw skeleton sequences as input and do not fully leverage the properties of dynamic hand gestures, whose most important clues are articulated movements of fingers and the global movements of the hand. To this end, we take advantage of hand-crafted motion features and deep features from RNN to promote the performance of dynamic hand gesture recognition from skeletal data.

In this paper, we propose a Motion Feature Augmented Network (MFA-Net) for skeleton-based dynamic hand gesture recognition. We extract finger articulated features from the hand skeleton by a variational autoencoder (VAE), which is an efficient and concise representation of the finger articulated movements. To describe the global movements of the hand, we extract the global rotation and global translation of the hand. A distance adaptive discretization scheme is exploited to better model the amplitude of the gestures. The finger motion features and global features along with the skeleton sequence are fed into a RNN to predict the class of input gesture. Experiments on the publicly available skeleton-based DHG-14/28 dataset [28] and SHREC’17 dataset [34] demonstrate the effectiveness of our proposed method.

A preliminary version of this paper is presented in [35]. This paper extends the preliminary work [35] in several aspects: (1) A more extensive survey on related work is provided, including RGB-D/skeleton based static/dynamic hand gesture and feature augmentation methods. (2) An improved feature representation is proposed to describe the configurations of hand skeleton articulation via variational autoencoder (VAE) and further promotes the performance on DHG-14/28 dataset. (3) The proposed method was evaluated on a new dataset (SHREC’17 dataset [34]) and outperformed state-of-the-art methods.

The remainder of this paper is organized as follows. In Section 2, we review prior approaches that are related to our proposed method. In Section 3, we present an overview about our proposed motion feature augmented network. In Section 4, we provide details of extracting motion features from hand skeleton sequences. Evaluations on public datasets and ablation studies are provided in Section 5. Section 6 gives a brief conclusion of this paper and discussion of future work.

## 2. Related Work

In this section, we briefly review recent methods on hand gesture recognition, which are broadly categorized into static hand gesture and dynamic hand gesture recognition. For dynamic hand gesture recognition, we briefly review related work on RGB-D based and skeleton-based methods. We also review some recent methods of feature augmentation. More comprehensive reviews on hand gesture recognition are found in [36,37,38,39].

### 2.1. Static Hand Gesture Recognition

Static hand gesture recognition aims to predict the gesture label for a single image. Ren et al. [15] proposed the finger earth mover’s distance metric (FEMD) for classifying hand gestures using Kinect camera. Wang et al. [40] proposed a new distance metric called superpixel earth mover’s distance metric (SP-EMD) to measure the dissimilarity between gestures. Chen et al. [3] first located the hand keypoints from the depth images and exploited angle features of finger roots for hand gesture recognition via finger length weighted Mahalanobis distance. Similarly, hand skeleton is estimated from depth image and joint angle features are used for classification in [4]. Koller et al. [41] exploited convolutional neural network (CNN) in weakly supervised training manner for hand gesture recognition. Despite its advancements in recent years, static hand gesture recognition fundamentally lacks of capability to handle temporal information and exhibits limitations for practical applications.

### 2.2. RGB-D Based Dynamic Hand Gesture Recognition

Dynamic hand gesture recognition from RGB-D frames has been actively researched for decades [42,43,44,45,46,47,48,49]. Zhu et al. [44] proposed a framework using 3D CNN and Convolutional LSTM to recognize gestures from both RGB and depth sequences. Molchanov et al. [6] proposed a recurrent 3D CNN to perform simultaneous dynamic hand gesture detection and classification from multimodal data, including depth, color, optical flow, and stereo IR streams. Zhang et al. [45] proposed a deep architecture to first learn spatiotemporal features using 3D CNN and bidirectional convolutional LSTM and learn higher-level features via 2D CNN. Köpüklü et al. [50] proposed a data level fusion strategy named Motion Fused Frames (MFFs) to fuse motion information into gesture sequences. However, RGB-D based dynamic hand gesture recognition may suffer from clustered background, heavy input data burden, etc.

### 2.3. Skeleton-Based Dynamic Hand Gesture Recognition

As the recent progress of fast and accurate hand pose estimation algorithms [18,19,20,21,22,23,24] and related sensors or cameras, 3D hand poses are more easily obtained. More research interests have been focused on skeleton-based dynamic hand gesture recognition.

De Smedt et al. [28] proposed a skeleton-based dynamic hand gesture recognition algorithm and suggested that skeleton-based method achieved superior performance over depth-based methods. In their approach, a new descriptor named Shape of Connected Joints (SoCJ) is encoded by Fisher vector representations to describe the hand skeleton. Histogram of hand directions and wrist orientations are adopted to represent the hand movements in global space. Temporal pyramid is exploited to model the temporal information. Similar representations are further exploited in [29] for hand gesture recognition via learning on Riemannian manifold. Boulahia et al. [30] adopted a feature set named Handwriting-Inspired Features (HIF3D) [51] which was originally proposed for skeleton-based action recognition to address the problem of skeleton-based dynamic hand gesture recognition.Caputo et al. [31] applied several processing methods (such as rotation, smoothing, scaling, etc.) on the hand gesture trajectory and matched it with templates using gesture distance metrics. These methods are based on carefully designed hand-crafted features, which may be not optimal for hand gesture recognition.

There are arising trends to use deep learning methods for skeleton-based dynamic hand gesture recognition. Núñez et al. [32] adopted the combination of CNN and LSTM for dynamic hand gesture recognition and action recognition from skeletal data. A two-stage training strategy is used to first train the CNN and then fine tune the whole CNN + LSTM network. Ma et al. [33] focused on addressing noisy skeleton sequences and proposed a LSTM network together with a nested interval unscented Kalman filter (UKF) to improve performance for noisy datasets.

Different from above existing methods, our proposed method takes advantages of both hand-crafted features and deep learning methods to obtain optimal features for hand gesture recognition.

### 2.4. Feature Augmented Method

There have been some attempts to enhance the capability of deep neural network by fusing hand-crafted features into the network. Sadanandan et al. [52] proposed the feature augmented deep neural networks that augmented the raw input images with eigen images to improve the performance of cell segmentation. Egede et al. [53] fused HOG features, geometric features and deep learned features into a Relevance Vector Regressor (RVR) to estimate pain intensity. Similarly, Manivannan et al. [54] concatenated hand-crafted features with CNN features for gland segmentation. Wang et al. [55] adopted the idea of combining hand-crafted features and CNN features to address the problem of action recognition.

Inspired by these methods, we exploited motion features to augment the neural network for better performance of skeleton-based dynamic hand gesture recognition.

## 3. Overview of the Proposed Framework

**Figure 1:**
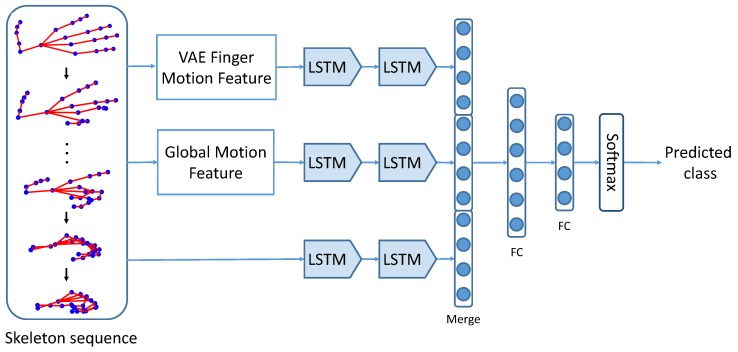
The framework of our proposed motion feature augmented network (MFA-Net). Finger motion features and global motion features are extracted from the input dynamic hand gesture skeleton sequence. These motion features, along with the skeleton sequence, are fed into different branches of a Long-Short Term Memory (LSTM) network to get the predicted class of input gesture.

The framework of our proposed motion feature augmented network (MFA-Net) is shown in Figure 1. MFA-Net takes a hand skeleton sequence as input and predicts the class label of dynamic hand gesture. It consists of three branches, which process finger motion features, global motion features and skeletons, respectively. The most important clues for a dynamic hand gesture are articulated movements of fingers and the global movements of the hand. Therefore, augmenting the original skeletons with finger and global motion features is beneficial to dynamic hand gesture recognition.

Firstly the global motion features and finger motion features are extracted from the input skeleton sequence. The global movement of a dynamic hand gesture can be represented by the global translation and rotation of the hand. For finger motion features, we explore two kinds of representations in this paper: kinematic features and variational autoencoder (VAE) features. The hand skeleton can be directly and effectively represented by a kinematic hand model whose parameters are the angles of bones, the global translation and global rotation [20,21]. Therefore, these kinematic hand parameters can serve as efficient and discriminating features for dynamic hand gesture recognition. We also explore latent features extracted from a hand skeleton by a variational autoencoder (VAE), which captures the latent representations of a hand pose. In our approach, theses features with offset and dynamic pose modeling are utilized as the motion features to represent dynamic hand gestures. The details of motion feature extraction are presented in Section 4.

We exploit the recurrent neural network (RNN) to model temporal information for its great successes in temporal sequences recognition tasks [6,56]. More specifically, we adopt the Long-Short Term Memory (LSTM) network, which is a successful variant of RNN that can model long temporal information of sequences. Although LSTM can somehow learn features from the input skeleton sequences, some information may be absent or weakened, which will hinder the classification performance. To this end, we augment features for LSTM by combining the global and finger motion features and the original skeleton. The finger motion features and global motion features are extracted from the input skeleton sequence. These motion features and the input skeleton sequence are fed into the LSTM. Each branch contains two LSTM layers and one fully connected (FC) layer. Outputs from three branches are concatenated together, followed by three FC layers and a softmax layer for class prediction. All layers are followed by a dropout layer and FC layers are followed by a ReLU function.

## 4. Motion Feature Extraction

In this section, we describe how to extract finger motion features H(S) and global motion features G(S) from the input hand skeleton sequence $S={\left\{s^{t}\right\}}_{t=1}^{T}$, where $s^{t}={\left\{x_{i}^{t},y_{i}^{t},z_{i}^{t}\right\}}_{i=1}^{J}$ denotes a hand skeleton for frame *t*, *T* is the number of frames of this sequence and *J* is the number of joints for hand skeleton.

### 4.1. Global Motion Feature

The global motion features (global rotation and global translation) are important for dynamic hand gesture. Typically, the global status of the hand can be determined by the wrist joint, palm joint and metacarpophalangeal (MCP) joints, which are denoted by $p^{t}$. We use Kabsch algorithm [57] to infer the global rotation $G_{r}$ and global translation $G_{l}$ of a hand skeleton:

(1) $$\left[G_{l},G_{r}\right]=Kabsch\left(p^{t},p_{0}\right),$$

where $G_{r}=\left(r_{x},r_{y},r_{z}\right)$ represents the rotations along three axes, $G_{l}=\left(\rho ,\theta ,\phi \right)$ is the spherical coordinate of global translation, $p_{0}$ is a reference palm that centers at (0,0,0) and faces the camera with the palm upwards.

The amplitudes of hand gestures differ from person to person for the same gesture. Therefore, previous work [28] ignored the amplitude part ρ of global translation. However, sometimes the amplitude is critical for gestures. For example, gesture Grab and gesture Pinch are quite similar except for the amplitude of the gesture. To this end, we propose a distance adaptive discretization (DAD) method to extract global translation amplitude feature, inspired by Distance Adaptive Scheme [4,58], which is used for feature selection for hand pose estimation. The DAD method discretizes ρ into *M* bins using the threshold ${\left\{\eta_{i}\right\}}_{i=1}^{M}$. A Gaussian distribution kernel g(x) is used to generate the thresholds:

(2) $$\int_{0}^{\eta_{i}}g\left(x\right)dx=\frac{i}{M}\int_{0}^{\sigma }g\left(x\right)dx,$$

where σ is the standard deviation of the Gaussian function. In our experiments, we set $\sigma =1.5r_{palm}$ where $r_{palm}$ is the radius of the palm. The global feature for a hand skeleton can be written as:

(3) $$\Phi^{t}=\left[\rho_{bin},\theta ,\phi ,r_{x},r_{y},r_{z}\right],$$

where $\rho_{bin}$ is the discrete representation of ρ using the thresholds determined by Equation (2).

Similar to previous work [59], we use offset pose $\Phi_{op}^{t}$ and dynamic pose $\Phi_{dp}^{t}$ for global features $\Phi^{t}$ to model the global motion features. The offset pose represents the offset from current global features to those of the first frame of gesture sequence:

(4) $$\Phi_{op}^{t}=\Phi^{t}-\Phi^{1}.$$

The dynamic pose represents the difference of global features between current frame and several previous frames:

(5) $$\Phi_{dp}^{t}=\left\{\Phi^{t}-\Phi^{t-s}|s=1,5,10\right\}.$$

There features can enhance the representability of the global motion of the hand and thus can model the temporal information of dynamic hand gesture. All above features are concatenated to form the global motion features $G^{t}\left(S\right)=\left[\Phi^{t},\Phi_{op}^{t},\Phi_{dp}^{t}\right]$ for frame *t*.

### 4.2. Finger Motion Feature

For finger motion features, we explore two kinds of representations, namely kinematic features and variational autoencoder (VAE) features, which are presented in Section 4.2.1 and Section 4.2.2, respectively. Kinematic finger motion features are exploited in our preliminary work [35] and in this paper we propose a more effective representation for finger motion feature using VAE. The impact of kinematic and VAE finger motion features is discussed in Section 5.3.

#### 4.2.1. Kinematic Finger Motion Feature

For many dynamic hand gestures, the articulated movements of fingers are critical because the global movements may be insignificant, especially for fine-grained gestures. We use 20 DoFs (degree of freedoms) to model the finger articulation movements. For the MCP joints, there are 2 DoFs for each joint. For proximal interphalangeal (PIP) and distal interphalangeal (DIP) joints, 1 DoF is used to describe the angle of bone. These kinematic parameters retain rich information for the shape of the hand skeleton. We use IK(·) to denote the inverse kinematics function that derives hand kinematic parameters from the original hand skeleton $s^{t}$:

(6) $$\Theta_{km}^{t}=\mathrm{IK}\left(s^{t}\right).$$

Similarly, we use dynamic pose $\Theta_{dp}^{t}$ and offset pose $\Theta_{op}^{t}$ to model the finger motion feature:

(7) $$\Theta_{op}^{t}=\Theta_{km}^{t}-\Theta_{km}^{1},$$

(8) $$\Theta_{dp}^{t}=\left\{\Theta_{km}^{t}-\Theta_{km}^{t-s}|s=1,5,10\right\}.$$

These features are concatenated to obtain the kinematic finger motion features $F_{km}^{t}\left(S\right)=\left[\Theta_{km}^{t},\Theta_{op}^{t},\Theta_{dp}^{t}\right]$ for frame *t*.

#### 4.2.2. VAE Finger Motion Feature

**Figure 2:**
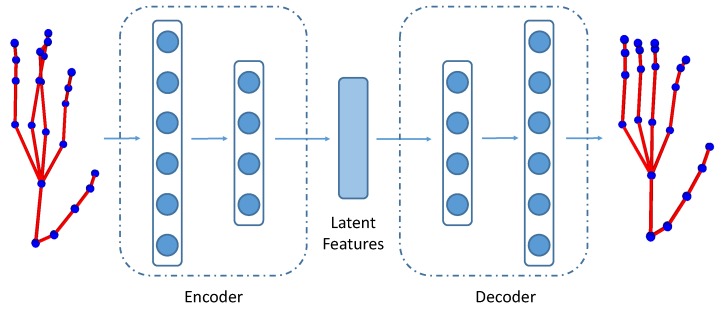
PoseVAE: Variational autoencoder for hand pose. We use the encoder to obtain latent features of the hand skeleton to describe the articulated movements of fingers.

The pose variational autoencoder (PoseVAE) consists of an encoder (Enc(·)) and a decoder (Dec(·)), as shown in Figure 2. Both the encoder and the decoder have two fully connected (FC) layers, with the dimensions of 32 and 20, respectively. The encoder projects the original hand skeleton into latent representations:

(9) $$\Theta_{vae}^{t}=Enc\left(s^{t}\right).$$

The decoder produces a decoded hand skeleton given the latent features:

(10) $$\tilde{s^{t}}=Dec\left(\Theta_{vae}^{t}\right).$$

The PoseVAE tries to minimize the distance between the original skeleton $s^{t}$ and the decoded skeleton $\tilde{s^{t}}$. We use the encoder to obtain latent features of the hand skeleton to describe the articulated movements of fingers. Similar to Equations (7) and (8), dynamic pose and offset pose are concatenated with the VAE features to obtain the VAE finger motion features $F_{vae}^{t}\left(S\right)$.

There are several benefits of using VAE features to represent the finger articulated motion. Firstly, learning latent representations for hand skeletons by PoseVAE has the potential to obtain more representative features than hand-crafted features such as kinematic features. Secondly, PoseVAE reduces the noises in hand skeletons that are introduced by inaccurate annotations. As shown in Figure 2, the input hand pose contains noise in the middle and ring fingers whose joints are inaccurately annotated, resulting in a physically implausible hand skeleton. The output pose of PoseVAE is much smoother and thus removes the unnecessary noise. Therefore, the latent representations learned by the PoseVAE are more robust and insensitive to the noise, which is beneficial to hand gesture recognition.

## 5. Experiments

In this section, we show the experimental results of our proposed method. Firstly, the datasets used for the experiments and some implementation details are briefly introduced. Secondly, comparisons with state-of-the-art methods and ablation studies are shown and discussed.

### 5.1. Implementation

The proposed framework was implemented in Keras [60]. We used Adam [61] algorithm with mini-batch of 32 to train the network. The parameters of Adam were set to the default settings suggested in [61], with learning rate lr=0.001, $\beta_{1}=0.9$, $\beta_{2}=0.999$ and $\epsilon =1\times 10^{-8}$. During training, the network minimized the cross entropy loss between the predicted labels and the ground truth labels. The network was trained for 100 epochs. In our experiments, *M* was set to M=5 in Equation (2). Every skeleton sequence was subtracted by the palm position of the first frame and scaled the amplitude to 1 before fed into third branch in Figure 1.

### 5.2. Comparison with State-Of-The-Art Methods

Although there are many dynamic hand gesture datasets, e.g. Chalearn IsoGD [49], Nvidia Gesture Dataset [6], etc., these datasets only provide RGB-D images and do not contains skeleton information. To evaluate our proposed method of skeleton-based dynamic hand gesture recognition, we conducted experiments on DHG-14/28 dataset [28] and SHREC’17 dataset [34], which provide hand skeleton annotations for each gesture sequence. We report the classification accuracy to evaluate our proposed method, which is the most commonly used evaluation metric for hand gesture recognition.

#### 5.2.1. DHG-14/28 Dataset

DHG-14/28 [28] is a public dynamic hand gesture dataset that provides hand gesture sequences with depth images and corresponding skeletons. The depth images were captured by Intel Realsense Camera and hand skeletons were obtained by Intel Realsense SDK. DHG-14/28 is a challenging dataset since it contains hand gesture from 20 subjects and has 14 gestures with two different finger configurations. Totally, DHG-14/28 consists of 2800 sequences. Each hand skeleton is represented by 22 joints. Since our proposed method focuses on dynamic hand gesture recognition from skeletal data, we only used the skeleton information of the datasets to conduct our experiments.

On DHG-14/28 dataset, we followed the same experimental setup as previous work [28,29,32,33,62,63,64], using the leave-one subject-out cross-validation (LOOCV) strategy for all following experiments. The proposed network was trained on data from 19 subjects and tested on the remaining one. Therefore, these experiments were repeated 20 times, with a different subject being used for testing. There are five fine-grained gestures and nine coarse gestures in DHG-14. In the experiments presented below, the MFA-Net was trained to classify 14 gestures and the classification accuracies of all gestures, five fine-grained gestures and nine coarse gestures are reported.

**Figure A1:**
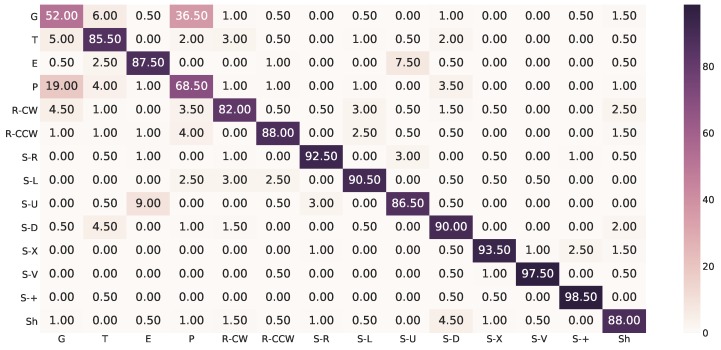
The confusion matrix of the proposed approach for DHG-14.

We compared our work with several state-of-the art methods [28,29,32,33,62,63,64] on DHG-14/28 dataset. The recognition rates of different methods on DHG-14 and DHG-28 dataset are shown in Table 1. It shows that our proposed method outperforms most state-of-the-art methods on DHG-14 dataset (14 gestures setting) and achieves comparable performance with SL-fusion-Average [64] and CNN + LSTM [32]. It should be noted that SL-fusion-Average [64] exploited both depth images and skeletons as input, while our method only relies on skeletons. CNN + LSTM [32], NIUKF-LSTM [33] and our proposed MFA-Net are all based on LSTM. NIUKF-LSTM [33] focuses on handling noisy skeleton data using nested interval unscented Kalman filter (NIUKF) and CNN + LSTM [32] utilizes CNN to learn spatiotemporal features from skeleton sequence. They both exploit LSTMs afterwards for gesture classification. Different from these work, our proposed MFA-Net focuses on augmenting the motion features for LSTM, which well preserves the properties of dynamic hand gestures. Moreover, the proposed MFA-Net can be compatible with existing LSTM-based methods by replacing the third branch of our method with prior methods (e.g., NIUKF-LSTM [33]). However, it is out of the focus of this paper to fully explore the combinations with prior work and we leave it for future work. To better illustrate the performance of our proposed algorithm, the confusion matrix of 14 classes is shown in Figure A1. It can be observed that the confusion between gesture *Grab* and *Pinch* is severe, due to the high similarity of these two gestures. However, our algorithm does improve the performance of these two gestures compared with those of SoCJ + HoHD + HoWR [28], with 60.25% average recognition rate for these two gestures of our method and 59.0% for SoCJ + HoHD + HoWR [28]. It can be observed that our method promotes the classification accuracy of fine-grained gestures and coarse gestures when compared with SoCJ + HoHD + HoWR [28].

**Figure A2:**
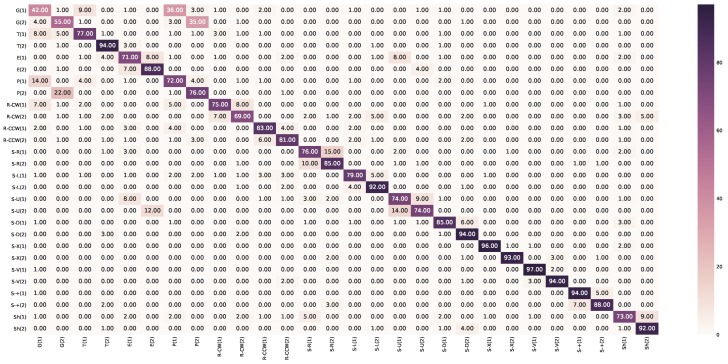
The confusion matrix of the proposed approach for DHG-28.

As shown in Table 1, our method is also better than most prior methods and achieves comparable performance with CNN + LSTM [32] when considering the more complicated 28-gesture classification task, which demonstrates the effectiveness of our proposed algorithm. Specifically, MFA-Nets boosts the accuracy by 6.85% when compared with a most recent method [64]. The confusion matrix of 28 classes is shown in Figure A2.

#### 5.2.2. SHREC’17 Dataset

SHREC’17 dataset [34] was first introduced in SHREC 2017 track to evaluate the performance of dynamic hand gesture recognition. Similar to DHG-14/28 dataset, SHREC’17 dataset also consists of 14 gestures performed by 28 participants executing the same gesture with two different configurations of fingers. There are 1960 sequences in the training set and another 840 sequences in the testing set.

Following the evaluation protocol of SHREC’17 track [34], we trained our MFA-Net on 1960 samples and evaluated on the other 840 samples, which is the same with previous work [28,30,31,34,65]. We followed a similar data augmentation strategy as in [32] to add random scaling, shifting, time interpolation and noise to the original sequences. After data augmentation, the whole training set contained 9800 samples.

As shown in Table 2, our proposed MFA-Net achieves the accuracy of 91.31% for 14 gestures and 86.55% for 28 gestures and outperforms all prior methods for both experimental settings. Specifically, MFA-Net improves the accuracy for 28 gestures by about 4.7% when compared with existing best performance by SoCJ + HoHD + HoWR [28] and 6.07% when compared with more recent work [30].

**Figure A3:**
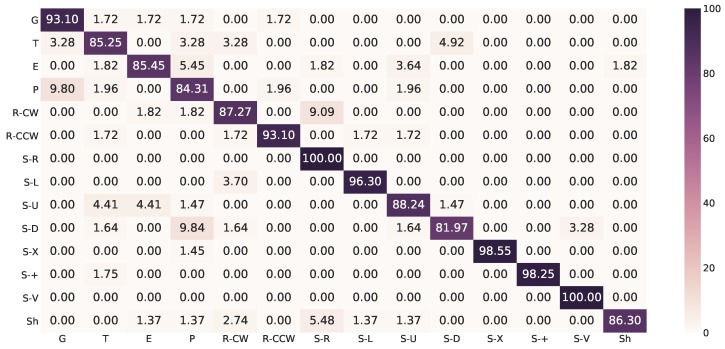
The confusion matrix of the proposed approach for SHREC’17 (14 gestures) dataset.

**Figure A4:**
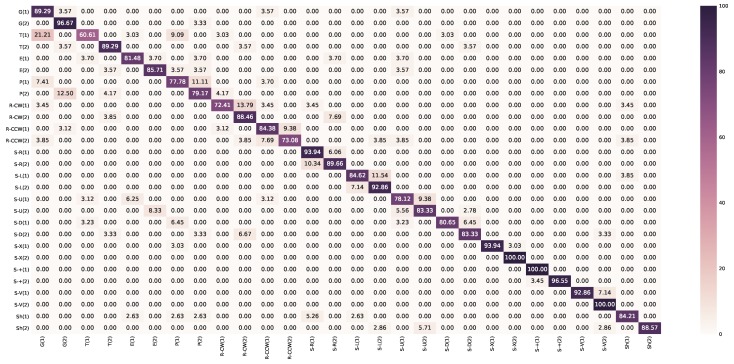
The confusion matrix of the proposed approach for SHREC’17 (28 gestures) dataset.

The confusion matrices for 14 gestures and 28 gestures recognition on SHREC’17 dataset are shown in Figure A3 and Figure A4, respectively. Our MFA-Nets achieves accuracy higher than 85.0% for 12 out of 14 gestures. For the more challenging 28 gestures task, MFA-Net obtains accuracy higher than 85.0% for 15 out of 28 gestures and accuracy higher than 80.0% for 22 out of 28 gestures.

### 5.3. Ablation Studies

To verify the contributions of different modules of our proposed method, we conducted several self-comparison experiments on DHG-14 dataset, which has 14 gestures.

Since DHG-14 exploits LOOCV strategy for evaluation, there are totally 20 splitting protocols for 20 subjects. Existing studies only report the average classification accuracy of these 20 experiments, which is not sufficient to evaluate the performance and robustness of hand gesture recognition algorithms for different participants. In the ablation studies, we report the worst, best and average results of 20 different splitting protocols as well as the standard deviation, which is a more comprehensive metric for taking the inter-subject effects into account.

#### 5.3.1. The Contributions of Motion Features Augmentation

We conducted several baseline experiments to explore how the motion feature augmentation strategy affects the accuracy of dynamic hand gesture recognition. The first baseline (*Skeleton*) only took the skeleton sequences as input and adopted LSTM for gesture recognition. The second baseline (*MF(Kinematic)*) only took motion features as input and removed the third branch of the framework shown in Figure 1. In this baseline, we exploited kinematic features for finger motion features.

As shown in the first three rows of Table 3, in most cases, combining skeleton and motion features outperforms two baselines in terms of worst, best, and average accuracy and the stand derivation. Overall, the *Skeleton + MF (Kinematic)* has better average accuracies for fine, coarse and all gestures, which verify the effectiveness of the proposed strategy of augmenting LSTM with motion features.

#### 5.3.2. The Contributions of VAE Features

We then explored the effects of using VAE features or kinematic features for finger motion feature extraction. Kinematic features were exploited in our preliminary version of MFA-Net [35] and it achieves accuracy of 84.69% for DHG-14 dataset, as also shown in the third row of Table 3. When using PoseVAE to extract latent representations to describe hand skeleton, the accuracy increases to 85.75%, as shown in the last row of Table 3. In addition, the accuracies for best and worse subject for all 14 gestures are also improved, which indicates the effectiveness of the VAE features.

#### 5.3.3. The Contributions of DAD Strategy

In Section 4.1, we introduce the distance adaptive discretization (DAD) strategy to handle with the amplitude of dynamic hand gesture. We conducted experiments to remove the term $\rho_{bin}$ produced by DAD strategy in Equation (3). As shown in Table 4, adding DAD term increases the accuracies in terms of worst, best, average accuracy and the stand derivation, which demonstrates the contributions of DAD method. Specifically, the overall accuracy increases from 84.60% to 85.75% for 14 gestures on DHG dataset.

#### 5.3.4. The Impacts of Different Classifiers

**Figure 3:**
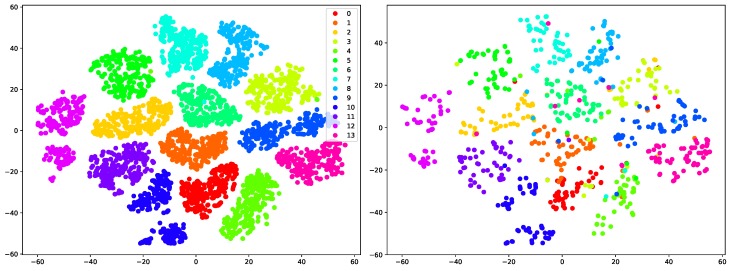
2D t-SNE visualization of features before FC layers: (**Left**) feature embeddings of training set on SHREC’17 dataset; and (**Right**) feature embeddings of testing set.

In the proposed MFA-Net, fully connected (FC) layers are utilized to classify gestures from deep features from LSTM blocks, as shown in Figure 1. To explore the impacts of different choices of classifiers and demonstrate the discriminability of the learned features, we extracted the deep features before the last two FC layers and fed them into different classifiers, including *k*-NN, an enhanced *k*-NN algorithm (Centroid Displacement-Based *k*-NN) [66] and random forest. The hyper-parameters for these classifiers were chosen using cross-validation on the training set. The recognition rates for different classifiers on SHREC’17 dataset [34] are shown in Table 5. One of the observations is that using FC layers as classifier performs better than others. It is intuitive since the deep features are learned together with the weights of FC layers. Another observation is that, even using simple classifier such as *k*-NN, the performances are good when compared with state-of-the-art methods. For example, the best prior performance on 14 gestures classification is 90.48% by Boulahia et al. [30], while *k*-NN obtains recognition rate of 90.60% and CD *k*-NN achieves 90.85%. On 28 gestures classification task, the highest recognition accuracy of existing methods is 81.90% [28], while both *k*-NN and CD *k*-NN achieve the accuracy of 86.07%. The considerably good performances of these classifiers demonstrate that the features produced by our proposed MFA-Net are quite discriminative for hand gesture recognition. To better understand this, we used t-SNE [67] to visualize the 2D embedding of the features. Figure 3 shows that the features of MFA-Net exhibit separable feature distributions in manifold and can be easily distinguished. Moreover, the feature embeddings on testing set are highly similar to those on training set, which is beneficial to obtain a good classifier.

### 5.4. Run Time Analysis

We evaluated the inference speed of MFA-Net on a computer equipped with 3.4 GHz i7-4770 CPU and Nvidia Tesla K40c GPU. MFA-Net takes about 1.47 ms to extract motion features for one hand skeleton and about 83.2 ms to predict the gesture for an input dynamic hand gesture sequence with 64 skeletons. Therefore, on average MFA-Net takes 2.77 ms to process one hand skeleton. In other words, MFA-Net can process 361 skeletons per second, which is sufficient for real-time performance.

## 6. Conclusions

This paper proposes the motion feature augmented network (MFA-Net) to recognize skeleton-based dynamic hand gestures. Finger motion features are extracted via a variational autoencoder from the hand skeleton sequence to describe finger articulated movements. Global motion features are utilized to represent the global movements of hand skeleton. The motion features, along with the skeleton sequence, are fed into three branches of RNN to predict the label of input gesture. Experiments demonstrate that our proposed MFA-Net achieves comparable performance with state-of-the-art methods on the public DHG-14/28 dataset and best performance on SHREC’17 dataset. Future work may focus on a hierarchical coarse to fine framework to achieve better classification performance.

## Figures and Tables

**Table 1:** Comparison of recognition rates (%) with state-of-the-art methods on DHG-14/28 dataset.

Method	DHG-14			DHG-28
	Fine	Coarse	Both	Both
HON4D [62]	-	-	75.53	74.03
HOG${}^{2}$ [63]	-	-	80.85	76.53
Smedt et al. [29]	-	-	82.50	68.11
SoCJ + HoHD + HoWR [28]	73.60	88.33	83.07	80.0
NIUKF-LSTM [33]	-	-	84.92	80.44
SL-fusion-Average [64]	76.00	90.72	85.46	74.19
CNN + LSTM [32]	**78.0**	89.8	85.6	**81.1**
**MFA-Net (Ours)**	75.60	**91.39**	**85.75**	81.04

**Table 2:** Comparison of recognition rates (%) with state-of-the-art methods on SHREC’17 dataset.

Method	14 Gestures	28 Gestures
HOD4D [62]	78.53	74.03
Riemannian Manifold [65]	79.61	62.00
Key Frames [34]	82.90	71.90
HOG${}^{2}$ [63]	83.85	76.53
SoCJ + HoHD + HoWR [28]	88.24	81.90
3 cent + OED + FAD [31]	89.52	-
Boulahia et al. [30]	90.48	80.48
**MFA-Net (Ours)**	**91.31**	**86.55**

**Table 3:** Recognition rates (%) of self-comparison experiments on DHG-14 dataset.

Method	Fine			Coarse			Both		
	**Best**	**Worst**	**Avg** ± **Std**	**Best**	**Worst**	**Avg** ± **Std**	**Best**	**Worst**	**Avg** ± **Std**
Skeleton	86.0	42.0	61.2 ± 12.37	97.78	74.44	86.44 ± 7.94	93.57	67.86	77.43 ± 6.82
MF(Kinematic)	84.0	46.0	71.5 ± 11.44	96.67	64.44	81.94 ± 8.17	90.0	58.57	78.21 ± 7.49
S + MF(Kinematic)	90.0	**56.0**	**76.9 ± 9.19**	97.78	72.22	89.0 ± 7.55	94.29	67.86	84.68 ± 6.67
S + MF(VAE)	**96.0**	48.0	75.6 ± 10.29	**100.0**	**76.67**	**91.39 ± 7.30**	**96.43**	**71.43**	**85.75 ± 6.71**

**Table 4:** Recognition rates (%) of MFA-Net with/without DAD strategy on DHG-14 dataset.

Method	Fine			Coarse			Both		
	Best	Worst	Avg ± Std	Best	Worst	Avg ± Std	Best	Worst	Avg ± Std
MFA-Net w/o DAD	92.0	42.0	74.2 ± 11.81	100.0	75.56	90.39 ± 6.89	**97.14**	67.86	84.60 ± 7.22
MFA-Net	**96.0**	**48.0**	**75.6 ± 10.29**	**100.0**	**76.67**	**91.39 ± 7.30**	96.43	**71.43**	**85.75 ± 6.71**

**Table 5:** Comparison of recognition rates (%) for different classifiers on SHREC’17 dataset.

Method	14 Gestures	28 Gestures
*k*-NN	90.60	86.07
CD *k*-NN [66]	90.83	86.07
Random Forest	90.36	85.24
**FC Layers (Ours)**	**91.31**	**86.55**

## Appendix A. Confusion Matrices

For DHG-14/28 [28] datasets, the confusion matrix of 14 classes is shown in Figure A1 and the confusion matrix of 28 classes is shown in Figure A2.

The confusion matrices for 14 gestures and 28 gestures recognition on SHREC’17 dataset [34] are shown in Figure A3 and Figure A4, respectively.
